# Original Translations

**Published:** 1884-02

**Authors:** 


### Original Translations.

ON THE TREATMENT OF EMPHYSEMA IN INFANCY.—BY DR. L. FURST, OF THE UNIVERSITY OF LEIPSIC, TRANSLATED FROM THE GERMAN, BY EDWARD SYDNEY MCKEE, M.D., CINCINNATI, OHIO, FROM GERHARDT'S HANDBUCH DER KINDER-KRANKHEITEN.

The pneumatic method may be described as altogether the most efficient in the treatment of the emphysema of adults of a grade not too far advanced. For reasons, however, which are to be sought in the difficulty with which are executed certain technical details, it may be said to be as yet far less efficient in the treatment of the same disease in children.

The difficulty, however, to which reference is made, is soon seen to be by no means formidable. Many of the obstacles it involves have already been surmounted; all promise to be soon levelled. When the technical details of the pneumatic method are more widely known and the results it can accomplish more generally recognized, it is safe to say that the time will arrive when as soon as an emphysema of the lung of a child is recognized, it can be relieved.

This strong assertion seems to be at variance with the view expressed by Vogel, that no diagnosis and therefore no therapy is possible in such cases. Certain it is that Vogel's view is not to be entertained in cases of simple alveolar ectasia without great tissue change.

Complete and true emphysema can not be forced to recovery, but here may yet be attained, through a judicious pneumatic treatment, a mitigation of symptoms and a lengthening of life—and such emphysema occurs seldom in children.

The difficulty of treatment in children lies in this, that in fact the process is not recognized as early as is necessary for restoration of the elasticity of the lungs. So that the more severe the primary trouble and the consequent injury to the organism of the lung thus rendered little capable of resistance, the greater the barriers to the success of the therapy here described.

Finally the awkwardness of little children, their restlessness and resistance to all mechanical treatment to which they are not accustomed, as also the difficulty of bringing them to exact, regular, productive in- and ex-piration, antagonize not a little the success of the treatment. From this we can get a hint that we cannot too early work prophylactically, especially in diseases which are wont to run into alveolar ectasia; and that on the other hand when we can make out lung emphysema, we dare lose no time in doing something for its relief. First for consideration is the indication to treat the primary trouble energetically, and above all with circumspection, to remove all hindrances to breathing, to remove catarrhal secretions and allay irritating coughs. These, it is well known, favor the origin of alveolar ectasia. But if such have already appeared, the indications are to direct treatment toward the disturbed relation between the inspiration, expiration and the lung capacity, and this alone on the plan of the pneumatic method. The numerous expectorant and emetic medicines hitherto employed for this purpose have never been followed by radical results, and only resulted in loss of time, while conditions, such as atelectasia, bronchial catarrh, etc., could have been in the same time improved by the pneumatic plan of treatment, and other cases complicated by difficulty of inspiration (croup, bronchitis capillaris, etc.) are remarkably improved to a great extent. In many cases a symptomatic treatment seems to suffice. In atelectasia from rapid unfolding of the embarrassed lung, in catarrh, in efforts to lessen secretion and to avoid the recurrence of laryngeal, tracheal and bronchial stenosis in croup, foreign bodies, etc., the fastest possible restoration of the passage of air in whooping cough and such spasms of coughing by the most careful management of the same, briefly, all primary troubles must react at the right time to favor the origin of alveolar ectasia. One must be on the lookout in pneumonia, in pleuritis of complete retrogression, and in every case of disease of the respiratory organs, and remember that a permanent emphysematous condition occurs as well in the lung regions concerned, as in those neighboring, if one does not prevent in time a lessening of the active lung capacity, an insufficiency of the respiration, and an urgent dyspnœa. But one can obtain that only through medical and dietetic treatment of the primary disease and frequent control of the lungs. When one finds deformity of the thorax, he must induce its removal as without this kind of early orthopædic treatment, emphysema is scarcely avoidable.

If one is not able to satisfy the indications of the disease, then he does well to treat the symptoms which are those most generally found in emphysema, viz.: the catarrh and cough.

First in view are the inhalations of alkalies (bicarbonate of soda and common salt), by means of an apparatus with double rubber qulbs. Inhalations of salt or of turpentine (ol. terebinthinæ) are followed by good success if practised in large inhaling saloons, pine forests, elevations, or on the sea shore; also alkaline and saline muriatic mineral water. The use of expectorants (infs. ipecac, liq. ammon. anis) from time to time, is peculiarly favorable to other cases. Cold spirituous bathing and rubbing of the neck, breast and back are praiseworthy, partly to promote the expectoration through reflex irritation, partly to fortify against new colds. Clean, temperate air, free from dust and smoke; protection from raw, dry winds; the maintenance of a regular warmth of the chest by wearing flannels, assist the removal of the catarrh. The irritating cough, when it exists without copious secretions, is removed by inspiration of steam, by inhalation of the infs. hyosciami, belladonnæ, stramonii; less surely by smoking saltpeter papers. At other times it is displaced by internal remedies, such as narcotics, aqua lauro-cerasi, morphia, essence of belladonna, etc. The over-pressure of the expiration, and the ever threatening disposition to emphysema, are lessened through the mitigation of the cough, by damp, mild, not decidedly fluctuating temperature, exceptional climate, especially in winter time, accompanied by a strong, digestible diet. If whooping-cough is the principal trouble, the best prophylactic against secondary emphysema, especially for tender children, is a changeable climate, and, indeed, not too pleasant, but somewhat damp and exhilarating.

An effectual therapy against the alveolar ectasia, or, in fact, against the emphysema, can only be possible when the same is formed. If the atrophy of the lung tissue has not yet commenced in any considerable degree, but the entire process is limited to loss of elasticity, then, and only then, can we hope to bring it to direct retraction, the emphysema having been not long in existence. The only rational way in which this end can be attained at present, is the pneumatic treatment which was founded by Hauke, perfected by Waldenburgh, Schmitzler, Lange, von Liebig, Biedert, and others. These gentlemen, in this department, have done no small service to therapeutics.

The function surface of the lungs is lessened by the accompanying dilatation. This continued and repeated dilatation must by and by injure the elasticity of the parenchyma, and later cause it to atrophy or disturb the nourishment. Here occurs the difficulty that one can, through absolute extension of the muscles of respiration, bring the lungs to retract completely, and, by enlarging the space for residual air, accomplish a complete ventilation.

As the air and secretions stagnate in the bronchioles, they again increase the ectasia. In children, in general, the vital capacity of the lungs is not great, also the expiration and thorax contraction are insufficient. Therefore they appear sunken and in need of treatment, which should be on the plan of Wintereich (compare Vol. I, p. 132, this work). The pneumatometer of Waldenburgh* shows little value when the children are old enough to make regular inspirations. In the normal state, the pneumatic pressure power is relatively high, perhaps for the greater movability and the greater elasticity of the thorax. On this account, according to Waldenburgh, it has less value than in grown persons.

The increased but not satisfied desire for air leads to dysnœa and after some time associates itself as a sequela. Sometimes occurs another deformity of the thorax, contracting thorax; further all the above named sequelæ on account of the practical sinking

* Original manufacturers Pölz & Flor, Berlin Unter den Linden, No. 14.

in or the partial bulging out. The pneumatic treatment accomplishes a direct improvement of all the appearances mentioned, now by rarefaction, now by compression of the air breathed. It applies to:

I. Compressed air to remove the insufficiency of inspiration, which is accomplished through an increase of the negative inspirations, pressure as well as the drawing in of a greater quantity of air.

II. Rarefied air sets aside the insufficiency of exspiration and increases the pressure of exspiration, so that a greater quantity of air is exhaled.

Both of the acts of breathing are assisted by regular changing and exact regulation of the pressure. These ends pneumatic cabinets on the one side, and pneumatic transportable apparatus on the other, have attempted to accomplish, but with very different success. The pneumatic cabinets, such as are found at Reichenhall, Nice, Ems, etc., are, indeed, baths in compressed air, into which the negative inspiratory air arises; and thereby more air is conducted into the lungs. Dilatation, of course, is favored as the patient experiences the wearisome expiration. To lighten this and raise the positive expiration pressure, this must be made into relatively thinner or absolutely thinned air. This innovation, at least through exhalation into atmospheric air, has already been introduced. This works in a doubtful manner, as Waldenburg has shown, on account of the great difference of pressure which is not capable of being regulated. Instead of this intensive and rapid dimunition of pressure one can on this account recommend only a gradual ascending pressure to promote the reaction of the lungs to which Kirauthe rightly gives the preference. Especially is this the case in the tender constitutions of children. These one should have exactly fixed in a pneumatic cabinet, and only then in behalf of the restitution of alveolar ectasia allow breathing. But in this sitting should be noticed not only the inhalation of compressed air, but also the grade of the rarefication of the atmosphere in which the expiration takes place. In other cases one should aim at the widening of the lungs with deep satisfying inspirations. The cure of chronic lung emphysema, however, is difficult. The pneumatic cabinets, if they are not made correctly and do not attain technical completeness, the exact regulation of the expiration pressure,resemble the transportable apparatus.

The transportable apparatus answered formerly in the high grade, because they made more promptly and more surely the exact regulation of the in- and ex-piration. Failure may be caused by the escape of a part of the air through the apparatus. The improved apparatus of Waldenburg[1], Schnitzler's modification of the same[2], Weil[3], and Biedert[4] are at present the only ones which can be thought of in reference to practicable usefulness, transportability and for rarefaction and compression of the atmosphere. All mention of anything similar to these are found in Knauthe's writings, or in Waldenburg and Biedert's works. In this work only so many are mentioned as are able to maintain a reliable, constant, thickness of the air for every phase of the respiration, and thus enable one exactly to decide in an individual case, when in a fitting manner to increase the disturbance of breathing. How far the alveolar ectasia of children extends to the grown is not a fixed fact. There is not yet a sufficient collection of data. According to Knauthe, the expiration into thinned air offers what fails in emphysema, since it is the "specific mechanical antidote" of Waldenburg, the proper remedy of Biedert. One in the future can only aim at the making of this important therapeutical aid available, not only with large intelligent, but also with little children. This is to be done through the greatest technical completeness possible.

Hanke, the meritorious originator of Pneumo-Therapy, calls attention to the fact, that the elasticity of a child's thorax furnishes a favorable condition, the narrowness of a child's air-passages an urgent indication. The cure of the difficulty by the self-regulating apparatus, brought to notice by him, has not been verified. The remedies discovered by Hanke have accomplished just as little at this stage of the experience. These are the pneumatic coat of mail (Wiener Med. Presse, 1874, No. 34 und

---

[1] Berlin, Windler, Dorotheen Str. 3.
[2] Vienna, of J. W. Hauck, Wieden, Kettenbrücke Gasse 20.
[3] Berlin, of Menter, Friedrich Str. 99.
[4] G. H. Jochem in Worms on the Rhein.

36) and the pneumatic tube[1] (Stricker's Jahrb. d. Med., Wien, 1877, I. vol., also Knauthe, l. c.) They are worthy of frequent trial, however, especially with small children.

These arrangements are very ingeniously contrived, the principal thing being without further assistance from the child to suitably regulate the pressure of air both without and within the lungs. They cover the body as well as the chest with a coat of iron. Within this, one can, at will, rarefy and compress the air so that in the one case the intra-pulmonic, in the other the extra-thoracic pressure predominates. One must acknowledge that such an arrangement, in case it proves practicable, may be made useful for little children, as the sittings cannot be rendered difficult by restlessness. It is only to be regretted that till now a regular alteration of the in- and ex-piration is not possible. One is, therefore, compelled for the present to continue both acts in rarefied atmosphere, then again draw a number of breaths of compressed air. Though in both cases disadvantages present themselves, they have some attainable uses. Strengthening of the ectasia, difficult expiration, dyspnœa, apnœa, disturbance of the circulation can occur in tender, young children, as Hauke himself has granted. The consolation subjoined by him, "that the child attains the satisfied feeling of a complete respiration only when it breathes constantly a succession of ascending respirations to the full dilatation of his lungs," has only limited power for good in some cases. These are atelectasia, laryngo- and broncho-stenosis through croup and rachitic thorax. So long as one cannot make the above mentioned indications satisfactory for the in- and ex-piration, such an apparatus does not work with success. It can only assist in lightening the inspiration, dyspnœa, and promoting the filling up of the lungs with oxygenated air. The above described transportable apparatus is used with good success with large children if the mouth pieces and masks fit exactly.

The cautions are :—

I. The sittings are not to be continued too long, nor to occur too often.

---

[1]Manufactured by Richard Mauch, Vienna, III., Apostel Gasse 14.

III. The difference in pressure dare be only gradually and cautiously increased and diminished.

III. The rarefied air is a powerful stimulant for the lungs. On this account are the acute inflammatory processes to be excluded from the pneumatic treatment.

The last point also forms a weighty indication against the pneumatic treatment in the related cases.

The curative action of the rational and thoroughly carried out pneumatic treatment is self-evident and lasting.

The inhalation of compressed air distends airless lungs and sunken thorax, increases the vital capacity and quiets the hunger for air. On this account, a richer supply of oxygen is made possible, a more thorough cleaning out of the carbonic acid gas, and a more decided exchange of gases. On the contrary, the exhalations into rarefied atmosphere cause quite an observable diminution of the lung volume, for the duration, even with stronger dilatation. The elasticity of the lungs and thorax can be compensated ; even the beginning disturbance of nutrition, for instance by capillary compression. The expectorations are lessened, and those severe symptoms of asthma and bronchitis stopped by the easing of the expirations. The exchange of gases in the expiration into rarefied atmosphere is more complete as the ventilation of the lungs becomes better, and the pent-up residual air is thrown out. Here are cited as further favorable results in pneumatic therapeutics and beginning emphysema: Improvement of the tissue change; the anæmia; the deformity of the thorax ; freedom of circulation ; cure of the accompanying catarrh, and lessening of the disposition to the same.

Pneumatic therapy has many successes to show for those special afflictions or complications which belong to emphysema. These complications are partly improved or cured as soon as the primary disease gives way. They are, asphyxia ; atelectasia, congenita et acquisita ; thorax rachitis ; insufflation ; dyspnœa in croup (Hauke) ; bronchitis ; asthma (Biermer); ectasia after whooping-cough, and bronchial catarrh (Lange, Waldenburgh von Liebig).

Here must yet be observed that Waldenburgh recommends in emphysema without bronchitis the asthma preponderating, the expiration into rarefied atmosphere; when bronchitis accompanies, inspirations of compressed air; in asthma the last during the attack, in the free intervals the usual in- and ex-pirations. Further, according to Langer, warm baths and cold douches assist very much the pneumatic treatment. The method of compression recommended by Gerhardt is suitable for many cases in which one can not arrange for a pneumatic apparatus. This satisfies the indications by mechanically narrowing the thoracic cavity, during the expiration, by enlarging the vital capacity and lightening the expectoration. One must carry out this compression of the elastic chest walls of children with caution and with careful consideration of cares and complications. Geyer recommends as a more constant substitute an elastic shirt. It is self-evident that this only increases the difficulty of inspiration.

The climatic treatment can only be directed against the complications and original affections. According to Biermann one shall, when he has bronchial catarrh, with dry, strong, cough and tenacious secretions, give the preference to low-lying, damp places for the time when the heat ranges 12 to 16 centigrade, especially so if there are sea coasts. It is not to be denied that the rarefied air of high elevations improves the ventilation of the lungs, lessens the insufficiency of the expiration and increases the contractility of the lung tissue. But the disadvantage of a high health resort for children more than for grown persons, lies in the permanence of the rarefaction of the surrounding air. In some cases severe consequences are sure to follow from the greater expansion of the gases of the blood oftener. The more tiresome and less deep the inspirations, which do not satisfy the hunger for oxygen, the more throwing out of water from the lungs, and the increase of the heart's action.

However tempting on first thought to advise a climatic cure for a child which has recovered from some severe disease, yet the correct choice of one which acts directly on the case is difficult.

It is understood from the first that one must in the treatment of emphysema combat the sequelæ as well as the constitutional troubles. These sequelæ are anæmia, intestinal troubles, chronic bronchial catarrh, asthma, and engorgement.

The therapeutics of emphysema of the cellular tissue, when interlobular, mediastinal or subpleural can not be discussed as it is not clinically diagnosticable. As symptomatic treatment of subcutaneous emphysema are recommended, compress bandages, dry cupping and punctures with the triocar.

BIBLIOGRAPHY.

Biedert, Ph. The Pneumatic Method and the Transportable Pneumatic Apparatus. Volkmmann's Klin Vortäge, No. 104, 1876.

Biermann, A. Climatic Health Resorts and Their Indications. Leipzig, 1872.

Hanke, J. An Apparatus for Artificial Respiration, etc. Vienna, 1870.

Knauthe, Th. Hand Book of Pneumatic Therapeutics. Leipzig, 1876.

Rilliet & Barthez. Treatment, Clinical and Practical of the Diseases of Children. Second edition. Paris, 1853. I.

Vievenot. Contributions to the Pneumatic Respiration Theory. Vienna, 1868.

Waldenburgh. The Local Treatment of the Diseases of the Respiratory Organs. Second edition. Berlin, 1872.

Ders. The Pneumatic Treatment of the Diseases of Respiration and Circulation, etc. Berlin, 1875.

---

PREGNANCY OF 56 YEARS.

Mr. Lapprey, making the autopsy of a woman 84 years of age, detected in the uterus a consistant tumor which was coverd by a stony layer. The woman had noticed the tumor first when she was 28 years of age, and it had never made her any other trouble than by its weight and volume. The physicians being present at the autopsy were astonished to find in the interior of the cyst a well-developed fœtus, from six to seven months old. By putting the fœtus accidentally in some strong solution it was destroyed. (The author does not say if the fœtus cried lustily after being 56 years in the uterus.—Tr.)—*Lyon Médical.*

